# The risk for rectal cancer recurrence and overall mortality is not increased in men previously diagnosed with prostate cancer: a report from the Swedish colorectal cancer registry

**DOI:** 10.1007/s00384-024-04710-y

**Published:** 2024-09-03

**Authors:** Ingvar Sverrisson, Kenneth Smedh, Abbas Chabok, Maziar Nikberg

**Affiliations:** Colorectal Unit, Västmanland’s Hospital, Department of Surgery and Centre for Clinical Research of Uppsala University, Västerås, Sweden

**Keywords:** Rectal cancer, Prostate cancer, Radiation therapy, Overall survival, Rectal cancer recurrence

## Abstract

**Introduction:**

Limited data exists on oncological outcomes following rectal cancer surgery in men who have previously been diagnosed with prostate cancer (PC). This study aimed to assess overall mortality and rectal cancer recurrence in men previously diagnosed with PC who underwent bowel resection.

**Methods:**

Data from the Swedish Colorectal Cancer Registry identified men who had rectal cancer surgery between 2000 and 2016, and the National Prostate Cancer Registry was used to identify those with a prior PC diagnosis. Cox regression analysis with propensity score matching was employed for data analysis. The primary outcome was overall mortality. Secondary outcome was recurrence for rectal cancer.

**Results:**

Out of 13,299 men undergoing bowel resection for rectal cancer between 2000 and 2016, 1130 had a history of PC. Overall mortality did not significantly differ between men with and without a prior PC diagnosis. Cox regression analyses with propensity score matching revealed that men with previously diagnosed low- or intermediate-risk (HR, 0.79; 95% CI, 0.70–0.90) and high-risk PC (HR, 0.85; 95% CI, 0.74–0.98) had lower overall mortality after rectal cancer surgery compared with men without a PC. There was no significant difference in rectal cancer recurrence between men with a previous low or intermediate-risk PC (HR, 0.92; 95% CI, 0.74–1.14) or high-risk PC (HR, 0.73; 95% CI, 0.52–1.01) compared with those without PC history.

**Conclusion:**

Men undergoing rectal cancer surgery with a previous diagnosis of prostate cancer do not experience an increased risk of rectal cancer recurrence or overall mortality compared with men without a previous history of prostate cancer.

**Supplementary Information:**

The online version contains supplementary material available at 10.1007/s00384-024-04710-y.

## Introduction

Prostate cancer (PC) and rectal cancer are two of the most common malignancies in men [1]. Radiotherapy (RT) has a well-established place in the treatment for both cancer types. For locally advanced rectal cancer, neo-adjuvant RT, either alone or in combination with chemotherapy, has been shown to decrease the risk for local recurrence and increase survival [2, 3]. Radiotherapy for PC can be used either alone or in combination with surgery and/or medical treatment to increase survival and improve outcomes [4–7]. However, radiotherapy in general, is associated with several adverse effects. Long-term adverse effects indicate that RT for pelvic malignancies increases the risk of developing rectal cancer later in life, while short-term effects of pelvic RT are mostly related to gastrointestinal and urogenital problems, as well as wound healing problems [8–10]. Due to the potential cumulative radiation dose toxicity and its adverse effects, neo-adjuvant RT is routinely not used in men with rectal cancer who have previously undergone irradiation for PC [11, 12]. Restricting the use of RT in these men not only increase the risk of more challenging surgery but can also have negative effects on local recurrence and survival after rectal cancer surgery. Data on prostate cancer treatment-related factors that might impact morbidity and oncological outcomes are limited. Some retrospective studies have indicated that previous radiotherapy for prostate cancer has a negative effect on post-operative morbidity, recurrence and survival after rectal cancer surgery [13, 14]. Additionally, hormonal therapy, surgical techniques, timing between treatments, patient comorbidities, radiation dose and field and genetic factors may also play significant roles in influencing these outcomes. Understanding the interplay of these factors is crucial for optimizing treatment strategies and improving patient prognosis. In our previous study on post-operative morbidity in men undergoing low anterior resection for rectal cancer, previously irradiated for PC, we identified a significantly lower risk of anastomotic leakage than was previously reported [13]. The aim of the current study is to analyse the overall survival and recurrence following bowel surgery for rectal cancer in men previously diagnosed with prostate cancer.

## Methods

Data was gathered from the Swedish Colorectal Cancer Registry (SCRCR) [14] and the National Prostate Cancer Registry (NPCR) [15]. Both registries prospectively collect data from all hospitals in Sweden, covering approximately 10 million inhabitants. In 2010, the NPCR was linked to a number of other population-based registries in Sweden, forming the Prostate Cancer Database (PCBaSe) [16]. This population-based retrospective cohort study included all men, older than 18 years, in the SCRCR who underwent resection for rectal cancer between 2000 and 2016. Among them, men with PC before their rectal cancer diagnosis were identified in the PCBaSe. Men who underwent emergency surgery were excluded. The following variables were analysed: age, stage of rectal cancer, treatment for PC, PC risk category, preoperative radiotherapy, preoperative radiochemotherapy, type of operation, level of rectal tumour, intraoperative bowel perforation, perioperative bleeding and local radical resection.

### Definitions

Rectal cancer was defined as an adenocarcinoma of the rectum located within 15 cm from the anal verge. The predominant neoadjuvant RT regimen during the study period for rectal cancer was short course RT (5 Gy five times over 1 week) followed by surgery. If a concomitant chemotherapy (CRT) was necessary, a long course of RT (1.8–2 Gy for 25–28 days over 6–8 weeks) was delivered with chemotherapy. According to Swedish national guidelines, MR of the rectum and CT examination of the chest and abdomen to detect metastases was recommended. Rectal cancer was classified at diagnosis according to tumour-node-metastases staging system (TNM 8th edition) [17].

PC was defined as an adenocarcinoma of the prostate. Low-risk PC was defined as T1–T2a tumours with Gleason score 6 and PSA < 10 ug/L, intermediate-risk PC was defined as T2b–T2c tumours and/or Gleason 7 and/or PSA 10–19 ug/L, and high-risk PC was defined as T3–T4 tumours and/or Gleason 8 + and/or PSA > 20 ug/L and metastatic PC as either “distant metastases” (defined as PSA ≥ 100 ug/L or M1) or “regionally metastatic” (defined as N1 and/or T4 and/or PSA 50–99 ug/mL). The most common curative RT regime for PC was external beam radiotherapy with a total dose of 78–80 Gy using 2 Gy/fraction.

Rectal cancer recurrence is defined as a recurrence of the disease, locally and/or distant metastases diagnosed with a computed tomography (CT) scan and/or a biopsy. Patients with rectal cancer are routinely followed up with a CT scan 1 and 3 years after the operation.

This manuscript is reported in accordance with the Strengthening the Reporting of Observational Studies in Epidemiology (STROBE) checklist for the reporting of observational studies [18].

### Statistical analysis

Patient demographics and baseline characteristics were summarized using descriptive statistics, medians and quartiles for continuous variables and percentages for categorical variables. For time-to-event endpoints, cumulative incidence curves defined as one minus the conditional survival function were calculated for mortality and recurrence, censoring at death. Imbalances in observed baseline variables were assessed and used to calculate propensity scores, to mitigate baseline imbalances between men without previous PC and men with different PC risk categories. The primary outcome was overall mortality, and secondary outcome was recurrence for rectal cancer. Cox regression models were employed to estimate adjusted hazard ratios for mortality and recurrence, including a variable for PC risk category with a separate category for men without previous PC, and propensity scores [19]. In supplemental analyses, a multivariable model was constructed with the propensity score matching variables as baseline covariates and also presented as stratified to preoperative radiotherapy. An unadjusted Cox regression model was also constructed, to assess the impact of propensity scores versus multivariable adjustment. A two-sided confidence level of 5% was set for all tests and confidence intervals, which were not adjusted for multiplicity. R version 4.1.1 was used for all statistical analyses.

## Results

The demographics and baseline characteristics are presented in Table 1. In total, 13,299 men underwent rectal cancer surgery between 2000 and 2016, with 56 individuals excluded due to emergency surgery. Among the remaining 13,243 men, 1130 (9%) were previously diagnosed with PC. Median age was 69 years, with men with a previous PC being older (73 years) than those without PC (69 years). Eighty-six percent of the men had stage 1–3 rectal cancer. The median distance of the tumour from the anal verge was 9 cm and did not differ significantly between men with and without previous PC. Neo-adjuvant RT was applied in 52% of the men with a higher prevalence among men without a previous PC diagnosis (53% vs. 38%). In men with a previous PC, 51% were operated with resection and permanent stoma, compared to 48% in men without previous PC. The median time between PC diagnosis and rectal cancer surgery was 52.5 months.

**Table 1:** Demographics and baseline characteristics of the study population

	No PC	Low- or intermediate-risk PC	High-risk PC	Metastatic PC	Missing risk category
Men	12113 (100)	548 (100)	277 (100)	209 (100)	96 (100)
Age (years)					
Median (IQR)	69 (61–76)	72 (67–77)	76 (71–81)	75 (69–80)	70 (65–77)
< 70	6335 (52)	207 (38)	55 (20)	55 (26)	45 (47)
70–74	2147 (18)	132 (24)	60 (22)	44 (21)	14 (15)
75 +	3629 (30)	209 (38)	162 (58)	110 (53)	37 (39)
Missing data	2 (0)	0 (0)	0 (0)	0 (0)	0 (0)
Received prostate cancer treatment					
Conservative treatment		203 (37)	60 (22)	17 (8)	33 (39)
Radical prostatectomy		44 (8)	6 (2)	3 (1)	2 (2)
RT		85 (16)	39 (14)	8 (4)	3 (4)
Radical prostatectomy and RT		1 (0)	1 (0)	0 (0)	0 (0)
Hormonal or surgical castration		57 (10)	123 (44)	164 (78)	18 (21)
Missing data		158 (29)	48 (17)	17 (8)	29 (34)
Rectal cancer stage (1–4)					
Stage 1–2	6450 (53)	316 (58)	144 (52)	103 (49)	58 (60)
Stage 3	3973 (33)	165 (30)	107 (39)	80 (38)	25 (26)
Stage 4	1383 (11)	51 (9)	19 (7)	23 (11)	10 (10)
Missing data	307 (3)	16 (3)	7 (3)	3 (1)	3 (3)
Preoperative RT					
No	5706 (47)	353 (64)	173 (62)	119 (57)	50 (52)
Yes	6407 (53)	195 (36)	104 (38)	90 (43)	46 (48)
Preoperative CRT					
No	10348 (85)	495 (90)	266 (96)	197 (94)	88 (92)
Yes	1765 (15)	53 (10)	11 (4)	12 (6)	8 (8)
Type of operation					
APE	4291 (36)	187 (34)	92 (33)	75 (36)	45 (47)
HP	1617 (13)	82 (15)	47 (17)	39 (19)	12 (12)
AR	6205 (51)	279 (51)	138 (50)	95 (45)	39 (41)
Level of rectal tumour (cm)					
0–5	2477 (20)	91 (17)	43 (16)	34 (16)	22 (23)
5–10	5883 (49)	264 (48)	140 (51)	99 (48)	51 (53)
> 10	3539 (29)	180 (33)	91 (33)	67 (32)	23 (24)
Missing data	214 (2)	13 (2)	3 (1)	9 (4)	0 (0)
Intraoperative colon/rectal perforation					
Yes	668 (5)	42 (8)	20 (7)	15 (7)	8 (8)
No	11333 (94)	500 (91)	254 (92)	192 (92)	86 (90)
Missing data	112 (1)	6 (1)	3 (1)	2 (1)	2 (2)
Perioperative bleeding (mL)					
< 500	6341 (52)	308 (56)	150 (54)	105 (50)	47 (49)
≥ 500	5772 (48)	240 (44)	127 (46)	104 (50)	49 (51)
Local radical resection					
Yes	11243 (93)	519 (95)	259 (94)	188 (90)	89 (93)
No*	813 (7)	28 (5)	18 (6)	19 (9)	6 (6)
Missing data	57 (0)	1 (0)	0 (0)	2 (1)	1 (1)

Low-risk prostate cancer was defined as T1–T2a tumours with Gleason score 6 and PSA < 10 ug/L. Intermediate-risk prostate cancer was defined as T2b–T2c tumours and/or Gleason 7 and/or PSA 10–19 ug/L. High-risk prostate cancer was defined as T3–T4 tumours and/or Gleason 8 + and/or PSA > 20 ug/L. Metastatic PC was defined as either “distant metastases” (defined as PSA ≥ 100 ug/mL or M1) or “regionally metastatic” (defined as N1 and/or T4 and/or PSA 50–99 ug/mL)

^*^Local radical resection is defined as “no” if it is uncertain or not evaluable

*APE* abdominoperineal excision, *CRT* chemoradiotherapy, *HP* Hartmann’s procedure, *IQR* interquartile range, *AR* anterior resection, *PC* prostate cancer, *RT* radiotherapy, *PSA* prostate-specific antigen

Rectal cancer is classified at diagnosis according to TNM 8 where stage 1 disease is without lymph node and distant metastases and the tumour does not invade outside of muscularis propria, stage 2 is without metastases but the tumour grows outside of muscularis propria, stage 3 has lymph node metastases but without distant metastases, and stage 4 has distant metastases

### Recurrence and survival

**Fig. 1:**
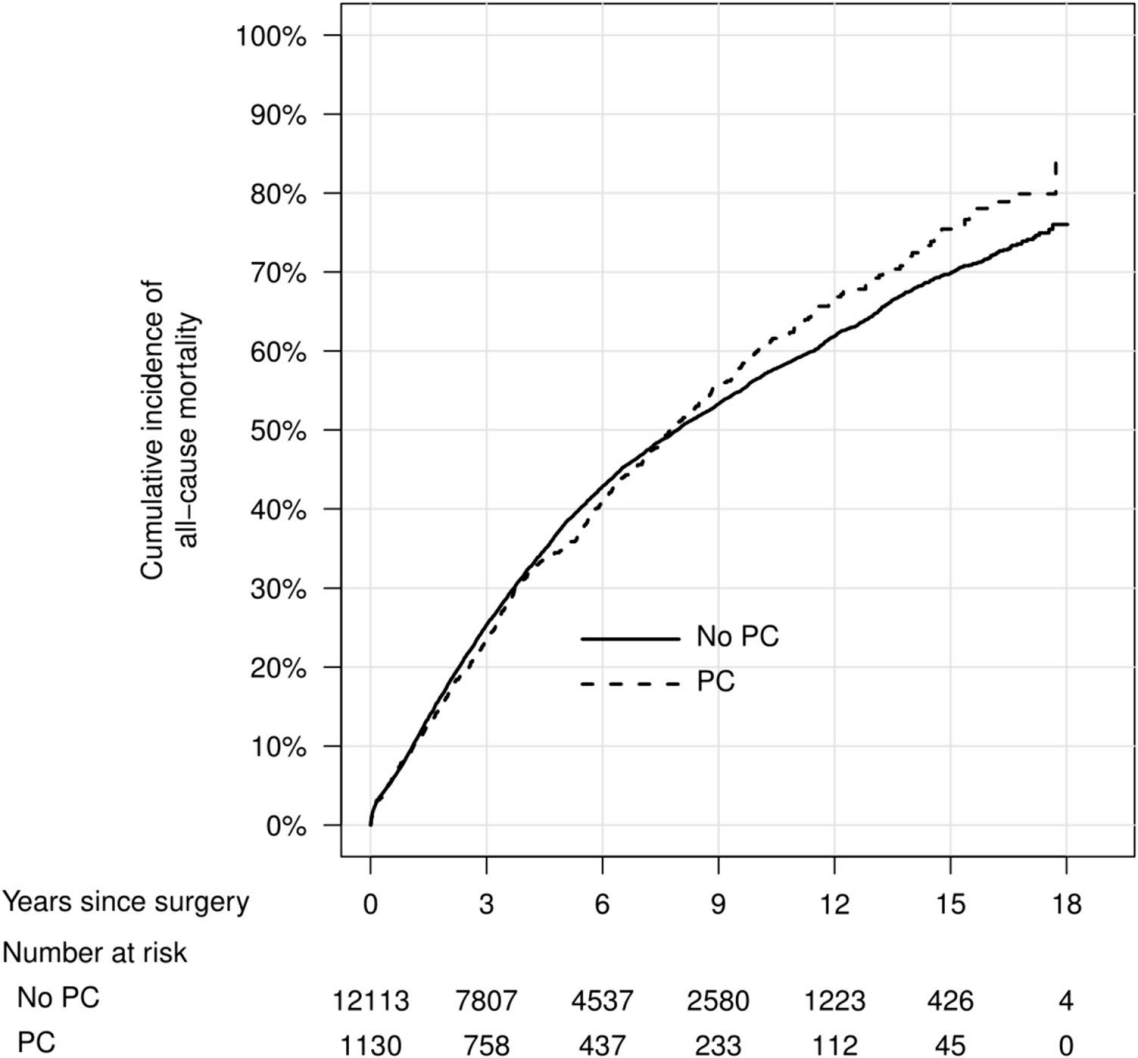
Cumulative incidence of overall mortality after rectal cancer surgery, in men with previous prostate cancer and men without previous prostate cancer. PC, prostate cancer

**Fig. 2:**
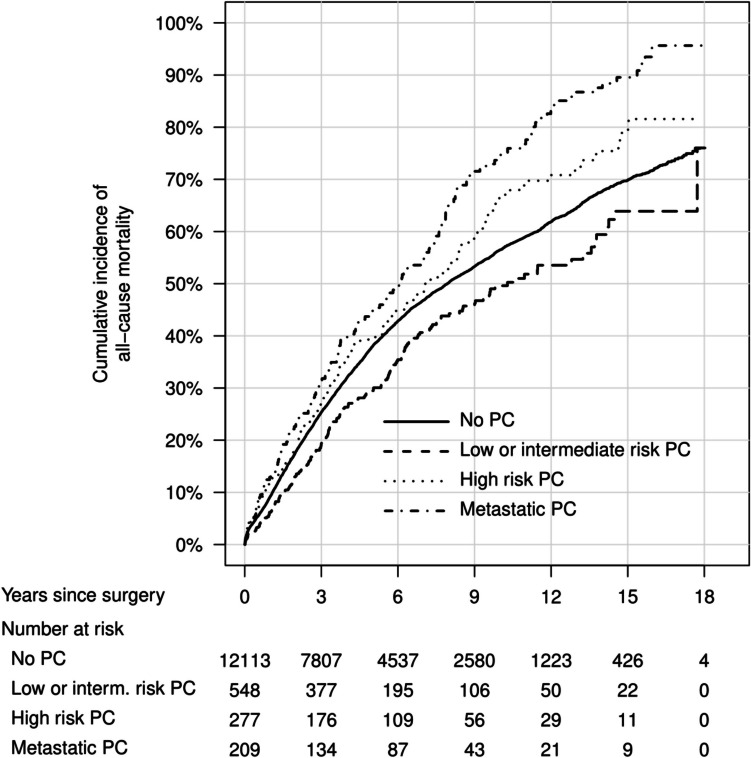
Cumulative incidence of all-cause mortality after rectal cancer surgery, in men with different prostate cancer risk categories and men without previous prostate cancer. PC, prostate cancer

**Fig. 3:**
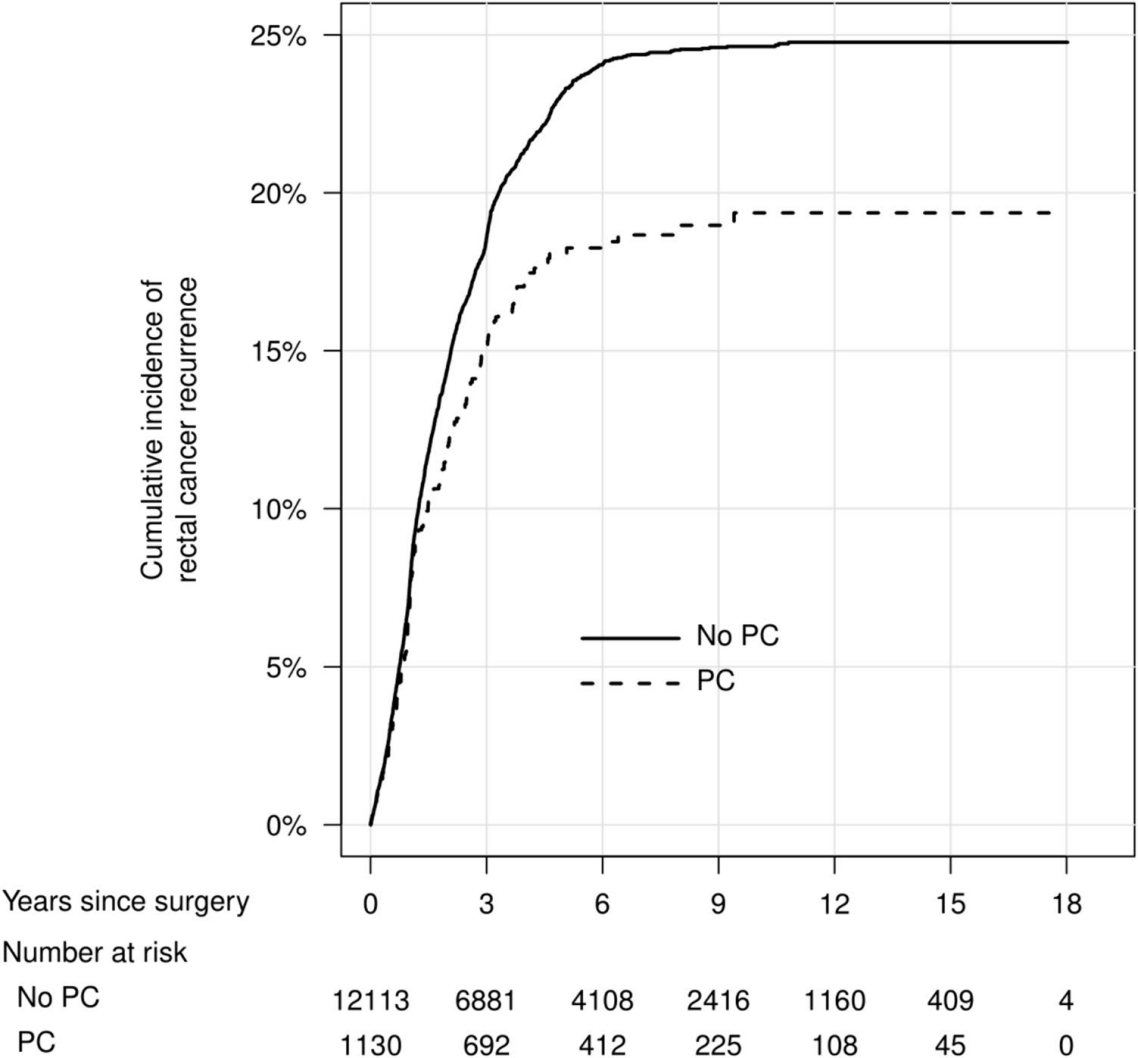
Cumulative incidence of rectal cancer recurrence after rectal cancer surgery, in men with previous prostate cancer and men without previous prostate cancer. PC, prostate cancer

**Fig. 4:**
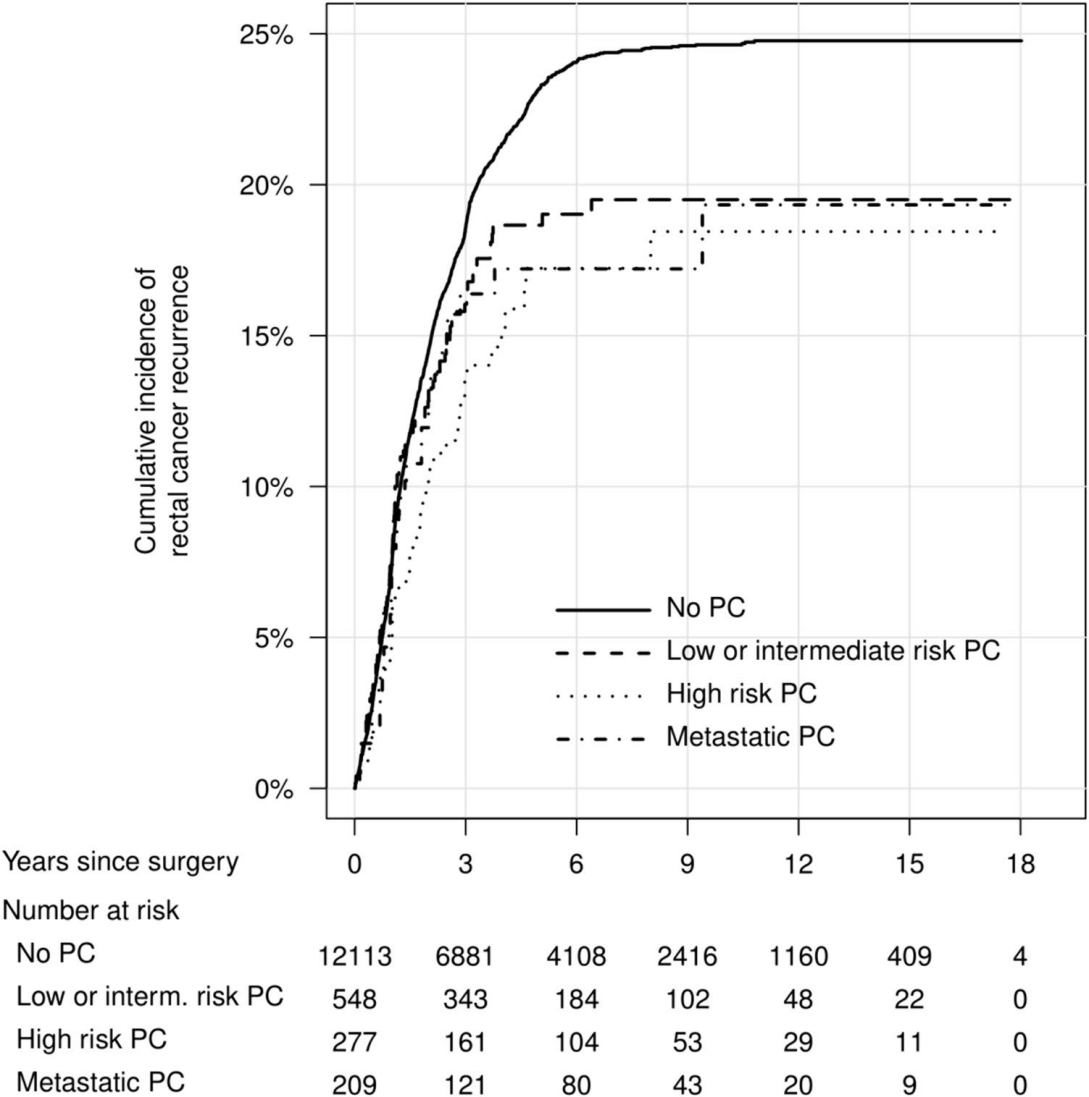
Cumulative incidence of rectal cancer recurrence after rectal cancer surgery, in men with different prostate cancer risk categories and men without previous prostate cancer. PC, prostate cancer

In cumulative incidence plots, overall mortality was similar between men with and without PC (Fig. 1), with the lowest mortality in men with low- or intermediate-risk PC (Fig. 2). The cumulative incidence of rectal cancer recurrence was highest in men without previous PC diagnosis (Figs. 3 and 4). The Kaplan–Meier figures stratified for radiotherapy are presented in Supplemental Figs. 1 and 2.

In Cox regression analysis, with propensity score matching, overall mortality was lower for men with low- or intermediate-risk PC (HR 0.79, 95% CI 0.70–0.90) and men with high-risk PC (HR 0.85, 95% CI 0.74–0.98), compared with men without PC. In an additional Cox regression analysis, with propensity score matching, stratified by preoperative RT, all-cause mortality for men with PC was lower (HR 0.87, 95% CI 0.79–0.97) in patients receiving perioperative radiotherapy compared with men without PC (Table 3). Men with metastatic PC also had lower risk of recurrence (HR 0.65, 95% CI 0.45–0.94) than those without a previous PC (Table 2). Additionally, men with PC not reviving preoperative radiotherapy had a lower risk of rectal cancer recurrence (HR 0.66, CI 0.50–0.87) compared with men with PC (Table 3). There were no statistical differences in patients receiving preoperative radiotherapy in relation to having had prostate cancer or not. The distribution of matching variables after propensity score matching is presented in Supplemental Table 1.

**Table 2:** Cox regression models for overall mortality and rectal cancer recurrence, with propensity score matching between men with previous prostate cancer and men without previous prostate cancer

	All-cause mortality				Rectal cancer recurrence			
	Subjects with events (%)	Event rate	HR	95% CI	Subjects with events (%)	Event rate	HR	95% CI
PC category								
No PC	3079 (62.6)	87.2	1.00	( Ref.)	874 (17.8)	33.8	1.00	( Ref.)
Low- or intermediate-risk PC	203 (38.7)	70.3	0.79	(0.70–0.90)	90 (17.2)	33.3	0.92	(0.74–1.14)
High-risk PC	150 (56.4)	103.0	0.85	(0.74–0.98)	38 (14.3)	27.5	0.73	(0.52–1.01)
Metastatic PC *	154 (76.2)	138.1	1.12	(0.99–1.28)	30 (14.9)	28.7	0.65	(0.45–0.94)

Event rates per 1000 patient years. Low-risk prostate cancer was defined as T1–T2a tumours with Gleason score 6 and PSA < 10 ug/L. Intermediate-risk prostate cancer was defined as T2b–T2c tumours and/or Gleason 7 and/or PSA 10–19 ug/L. High-risk prostate cancer was defined as T3–T4 tumours and/or Gleason 8 + and/or PSA > 20 ug/L. Metastatic PC was defined as either “distant metastases” (defined as PSA ≥ 100 ug/mL or M1) or “regionally metastatic” (defined as N1 and/or T4 and/or PSA 50–99 ug/mL). The following variables were included in the propensity score matching: age at index bowl resection, rectal cancer stage, preoperative RT, preoperative CRT, intraoperative colon/rectal perforation, perioperative bleeding (< 500 vs. ≥ 500 mL), and local radical resection

*CI* confidence interval, *HR* hazard ratio, *PC* prostate cancer, *PSA* prostate-specific antigen, *RT* radiotherapy, *CRT* chemoradiotherapy

**Table 3:** Cox regression model, with propensity score matching, stratified by preoperative RT

	All-cause mortality				Rectal cancer recurrence			
	Subjects with events (%)	Event rate	HR	95% CI	Subjects with events (%)	Event rate	HR	95% CI
No preoperative RT								
No PC	1960 (55.5)	90.1	1.00	( Ref.)	748 (21.2)	37.2	1.00	( Ref.)
PC	199 (53.5)	85.5	0.91	(0.81–1.03)	55 (14.8)	24.9	0.66	(0.50–0.87)
Preoperative RT								
No PC	2170 (52.8)	104.2	1.00	( Ref.)	702 (17.1)	36.1	1.00	( Ref.)
PC	307 (49.7)	98.2	0.87	(0.79–0.97)	102 (16.5)	35.0	0.90	(0.73–1.11)

Event rates per 1000 patient years. Low-risk prostate cancer was defined as T1–T2a tumours, with Gleason score 6 and PSA < 10 ug/L. Intermediate-risk prostate cancer was defined as T2b–T2c tumours and/or Gleason 7 and/or PSA 10–19 ug/L. High risk prostate cancer was defined as T3–T4 tumours and/or Gleason 8 + and/or PSA > 20 ug/L. Metastatic PC was defined as either “distant metastases” (defined as PSA ≥ 100 ug/mL or M1) or “regionally metastatic” (defined as N1 and/or T4 and/or PSA 50–99 ug/mL). The following variables were included in the propensity score matching: age at index bowl resection, rectal cancer stage, preoperative RT, preoperative CRT, intraoperative colon/rectal perforation, perioperative bleeding (< 500 vs. ≥ 500 mL), and local radical resection

*CI* confidence interval, *HR* hazard ratio, *PC* prostate cancer, *PSA* prostate-specific antigen, *RT* radiotherapy, *CRT* chemoradiotherapy

Similar estimates were obtained when baseline variables were included in multivariable Cox models instead of propensity scores (Supplemental Table 2).

## Discussion

In this population-based study utilizing data from two national registries, men who underwent rectal cancer surgery with a history of PC lived longer and had fewer rectal cancer recurrences compared with those without a prior PC diagnosis. This trend was observed for both low or intermediate- and high-risk prostate cancer. Notably, the majority of these patients had non-restorative surgery, and surprisingly, the frequency of permanent stoma did not differ between men with and without a previous PC diagnosis.

This contradicts results from several recent cohort studies. Guandalino et al. (2015) reported data on post-operative outcomes of 84 men with mid or low rectal cancer, including 8 who had received external beam RT (EBRT) for prostate cancer. Their multivariate analysis indicated that EBRT significantly decreased the overall survival (OS) and local recurrence free survival after rectal cancer surgery [20]. In a study from 2018, Feinberg et al. reported data of 7096 men who underwent rectal cancer surgery. Of these, 229 had a history of PC with worse 5-year OS rates in men with a history of PC with or without RT [21]. Lakis et al. (2019) reported data from the French GRECCAR group, comparing men with rectal cancer with and without previous PC treatment. Their results show significant increase in local recurrence and worse disease-free survival and overall survival for men with previous PC treatment [22].

The present study is the largest to date on the topic, including over 13,000 men treated for rectal cancer, of whom 1130 had a previous PC diagnosis. A previous study on post-operative complications in the current cohort, published in 2018, found a lower frequency of anastomotic insufficiency in men previously treated with RT for PC compared to previous studies. Additionally, there were no significant difference in 30-day re-operation, overall complications or surgical complications between those who received RT for PC and those who received RT for rectal cancer [13]. These results are consistent with the current findings, indicating comparable overall survival for men with non-metastatic history of PC and similar or lower risk of rectal cancer recurrence compared to men without a prior PC diagnosis.

As expected, fewer men with a previous PC diagnosis received neoadjuvant RT, and the rectal cancer recurrence was even lower in men with previous PC where radiotherapy was omitted compared with men with no PC. The improved overall survival in rectal cancer patients with a history of PC may be explained by selection bias, where men fit enough to survive PC and its treatment, live long enough to develop rectal cancer and remain healthy enough to qualify for surgery with bowel resection, which are likely to be the fittest individuals in the PC group. The improved survival even with respect to previous PC diagnosis might also be reflected by the nationwide implementation of TME surgery and centralization of rectal cancer surgery in Sweden [23, 24].

The analysis was not stratified by PC treatment due to incomplete data on RT for PC in the early years of the register. Instead, the analysis was stratified by PC risk category, where intermediate-risk PC and high-risk PC were used as proxies, as 19% and 25%, respectively, of men in these categories receive RT as a standard treatment, and the majority received external beam radiotherapy with a smaller proportion receiving brachytherapy [7]. Unfortunately, we did not have access to cancer-specific mortality data. However, there was no difference in rectal cancer recurrence in patients having preoperative radiotherapy with respect to previous PC diagnosis, and the recurrence rate was even improved in patients who did not receive preoperative radiotherapy and had a diagnosis of PC compared with those with no PC diagnosis. This strengthens the findings that a prior PC diagnosis does not have a significant negative impact on recurrence rates in this cohort.

Strengths of this study include the combination of two nationwide high-quality registers including more than 1100 men with rectal cancer previously treated for PC, and the propensity score matching process effectively balanced the baseline characteristics between the groups. Additionally, the SCRCR demonstrates high validity in registering recurrences and maintains a complete registration of all death dates. Limitations include the lack of cancer-specific data analysis, the potential risk for selection bias and residual confounding and small numbers in some PC risk categories. The impact of selection on generalizability of results, indicated by an imbalance in distribution of baseline variables, should be considered. Furthermore, the variabilities in radiotherapy treatment modalities, such as the proportion of patients receiving external beam radiotherapy versus brachytherapy, could influence the observed outcomes and warrants further investigation. The results must be interpreted with the understanding that the proportion of patients undergoing abdominoperineal excision in Sweden during the study period was approximately 35%, regardless of whether patients had a prior PC diagnosis or not.

With an ageing population and an increasing incidence of cancer in younger population, the likelihood of encountering men who develop both prostate and rectal cancers is expected to rise. This underscores the importance of this work in the clinical decision-making when managing these individuals.

## Conclusion

The data from this nationwide study indicates that men undergoing rectal cancer surgery with a previous diagnosis of prostate cancer do not experience an increased risk of rectal cancer recurrence or overall mortality compared with men without a previous history of prostate cancer.

## Supplementary Information

Below is the link to the electronic supplementary material.

Supplementary file1 (DOCX 31 KB)

## Data Availability

The data that was used and analysed for this study are available on request from the corresponding author.

## Declarations

### Declaration of AI and AI-assisted technologies in scientific writing

None declared
